# Impact of GnRH ovarian stimulation protocols on intracytoplasmic sperm injection outcomes

**DOI:** 10.1186/1477-7827-7-5

**Published:** 2009-01-15

**Authors:** Fátima Pinto, Cristiano Oliveira, Margarida F Cardoso, José Teixeira-da-Silva, Joaquina Silva, Mário Sousa, Alberto Barros

**Affiliations:** 1 Faculty of Engineering, University of Porto, Porto, Portugal; 2 Centre for Reproductive Genetics A. Barros, Porto, Portugal; 3 Department of Population Studies, ICBAS-Institute of Biomedical Sciences Abel Salazar, University of Porto, Porto, Portugal; 4 Lab Cell Biology, ICBAS-Institute of Biomedical Sciences Abel Salazar, University of Porto, Porto, Portugal; 5 Department of Genetics, Faculty of Medicine, University of Porto, Porto, Portugal

## Abstract

**Background:**

Although a large number of studies have been conducted in relation to ovarian response and pregnancy after GnRH agonist and GnRH antagonist controlled ovarian hyperstimulation protocols, most of them used single or combinations of a few predictive factors, and none included the stimulation protocol in the multivariable analysis. The present study was thus primarily designed to investigate the predictive value of the stimulation protocol and to analyze the possible relationships between stimulation protocols and treatment outcomes after adjusting for a large set of variables that potentially affect reproductive outcomes. Factors related to pregnancy achievement and predictive of the number of oocytes retrieved and high quality of the embryos obtained were also analyzed.

**Methods:**

To analyze the impact of GnRH ovarian stimulation protocols on the independent predictors of ovarian response, high quality embryos and clinical pregnancy, two groups out of 278 ICSI treatment cycles were compared prospectively, 123 with a GnRH agonist and 155 with a GnRH antagonist, with multivariable analysis assessing outcomes after adjusting for a large set of variables.

**Results:**

Antagonists were significantly associated with lower length and total dose of GnRH, lower length of rFSH, and higher numbers of oocytes and high quality embryos, whereas the agonist presented a higher fertilization rate and probability of pregnancy. Significant predictors of retrieved oocytes and high quality embryos were the antagonist protocol, lower female age, lower serum levels of basal FSH and higher total number of antral follicles. Significant predictors of clinical pregnancy were the agonist protocol, reduced number of attempts, increased endometrial thickness and lower female age. The probability of pregnancy increased until 30 years-old, with a decline after that age and with a sharp decline after 40 years-old.

**Conclusion:**

The models found suggest that not only the protocol but also factors as female age, basal FSH, antral follicles, number of attempts and endometrial thickness should be analyzed for counselling patients undergoing an ICSI treatment.

## Background

The most common ovarian stimulation regimens presently used are those employing gonadotropin-releasing hormone (GnRH) agonists or antagonists to prevent a premature LH surge [1]. Although there is controversial discussion about the better regimen choice [2], clinical advantages of GnRH antagonists over agonists are the absence of the initial stimulation gonadotropin release (flare-up effect) and, as a consequence, a more direct, immediate and reversible suppression of gonadotropin secretion by blocking the GnRH receptor, which allows their use without the need for a desensitization period [3]. Multicenter, randomized, prospective studies also revealed that exposure to GnRH antagonists is shorter and that the amount of exogenous gonadotropins needed as well the occurrence of ovarian hyperstimulation syndrome (OHSS) is reduced. Although patients using the antagonist regimen had lower number of oocytes and embryos, the percentage of mature oocytes and the fertilization and pregnancy rates were identical in both groups [1,3-9]. However, meta-analyses have led to discordant conclusions, showing lower implantation and pregnancy rates with antagonists [10-13], or no significant differences between the two protocols regarding prevention of the premature LH surge and occurrence of OHSS [12], or the probability of live birth [14]. Some authors thus suggested that the purpose of GnRH analogues can be reached either by a long agonist protocol or an oral contraceptive pretreated fixed antagonist protocol [15].

Several studies have been performed to identify predictors of ovarian response, such as female age, ovarian volume, number of antral follicles, ovarian stromal blood flow, serum FSH, LH, estradiol and inhibin B, cigarette smoking and body mass index. Similarly, predictors of pregnancy achievement were studied regarding female age, serum FSH, estradiol and inhibin B, ovarian volume, endometrial thickness, embryo quality, smoking status, body mass index and parity [16-33].

Although these studies have been conducted in relation to ovarian response and pregnancy, most of them used single or combinations of a few predictive factors, and none included the stimulation protocol in the multivariable analysis. The present study was thus primarily designed to investigate the predictive value of the stimulation protocol and to analyze the possible relationships between stimulation protocols and treatment outcomes after adjusting for a large set of variables that potentially affect reproductive outcomes. Factors related to pregnancy achievement and predictive of the number of oocytes retrieved and high quality of the embryos obtained were also analyzed.

## Methods

### Patients

Under informed consent, a total of 278 women were included. They were among those undergoing controlled ovarian hyperstimulation with a GnRH agonist or a GnRH antagonist protocol for an infertility treatment ICSI cycle. Women starting an infertility treatment ICSI cycle were followed forward in time towards the results of treatment. To use comparable groups of women, data was collected by physicians with a large experience in reproductive medicine based on the ovarian stimulation protocols routinely used in two different years and not based on a clinical judgement made by the physician in accordance with the patient's response in previous attempts. All data was obtained by the same team, reducing the variability related to measurement due to different observers with different practices. For all women, the number of previous attempts was considered and for cases that underwent more than one embryo transfer only the last cycle was included. Criteria for inclusion were: both ovaries present, with no morphological abnormalities; normal ovulatory cycle (25–35 days); basal FSH (day 3) serum level < 10 mIU/mL; no history of poor ovarian response; and a body mass index of 18–27 kg/m^2^. All patients and partners had a normal karyotype.

### Controlled ovarian hyperstimulation protocols

For the GnRH agonist long protocol (n = 123), buserelin acetate (0.6 mg/d, sc; Suprefact: Hoechst, Frankfurt, Germany) was started in the mid-luteal phase of the previous cycle. After pituitary down-regulation, the dose was reduced to 0.4 mg/d, and recombinant FSH (rFSH; Gonal F: Serono, Geneve, Switzerland; Puregon: Organon, Oss, The Netherlands) was added until either the leading follicle reached a mean diameter of 18 mm or two or more follicles reached a diameter of 17 mm. For the GnRH antagonist multiple-dose flexible protocol (n = 155), rFSH was started on the 2nd or 3rd menstrual-cycle day (with or without previous oral contraceptive pretreatment). The GnRH antagonist cetrolelix (Cetrotide: Serono) or ganirelix (Orgalutran: Organon), 0.25 mg/d, sc, was added daily, starting when the leading follicle reached a diameter of 12 mm and until either the leading follicle reached a mean diameter of 18 mm or two or more follicles reached a diameter of 17 mm. For both protocols, the initial rFSH dose was chosen according to the female characteristics, with no significant differences being observed between patients allocated to the agonist or the antagonists groups. Starting doses of rFSH ranged between 100–185 IU, 200–250 IU and 300–450 IU. Independently of the protocol to which patients were submitted, higher rFSH starting doses were associated with increased female age, basal FSH, body mass index and number of previous attempts, and with decreased ovarian volume and total number of antral follicles. For both protocols, urinary hCG (5,000–10,000 IU, im; Pregnyl: Organon) was administered 35 h before recovery of large ovarian follicles by ultrasonically-guided follicular aspiration, using flush medium (Medicult, Copenhagen, Denmark). Embryo transfer was performed under ultrasonography. All patients had luteal supplementation with three times daily intravaginal administration of 200 mg natural-micronized progesterone (Utrogestan, Jaba, Berlin, Germany). Implantation was confirmed by a rise in serum β-hCG on days 12 and 14 after embryo transfer. A clinical pregnancy was established by ultrasonography at 5–7 weeks of gestation. All couples agreed to have an integrated biochemical screening (at 12 and 15 weeks) and prenatal diagnosis in case of a positive test.

### Laboratorial procedures

Ejaculates were submitted to gradient centrifugation (Suprasperm System, Medicult), washed and incubated (5% CO_2_, 37°C, in filtrated humidified atmosphere) in sperm preparation medium (SPM; Medicult) to collect the swim-up fraction. Oocytes were denuded enzymatically (Synvitro Hyadase, Medicult) and mechanically (SweMed, Frolunda, Sweden). After culture in IVF medium (Medicult) for 2 h, they were microinjected as described in SPM [34]. They were then cultured in ISM1 medium (Medicult) for 2 days and then changed to ISM2 (Medicult). Normal fertilization was assessed 14–18 hours after injection, and embryo quality was evaluated according to the number, size and regularity of the blastomeres, and the percentage of fragments (high quality embryos were those with the correct number of cells, with similar size and regularity, and with less than 25% of fragments). Embryos were frozen with Embryo Freezing Medium (day 3) or with Blast Freeze (day 5) (Medicult) in an automatic freezing apparatus (Planer, Kryo 10 Series III).

### Statistical analysis

For a characterization of the study population, a descriptive analysis was done. Statistical analyses were performed using chi-square test (with Yates's continuity correction), Student's t-test and Mann-Whitney test as appropriate. In order to investigate possible predictors of ovarian response, the first stage of the analysis was to establish which of the factors were significantly associated with ovarian responsiveness. The end point was the number of oocytes retrieved and of embryos with high morphological quality. Statistical analysis was performed using univariable linear regression analysis, with the number of oocytes and embryos as dependent variables, to determine which variables predicted the outcomes. Multiple regression analysis by least-squares regression was then used to evaluate the predictive values of the different parameters in a stepwise manner. All predictive variables were entered the model as independent variables: age, type of protocol, basal estradiol and FSH levels, number of antral follicles, ovarian volume, body mass index (BMI), smoking status, total number of attempts and cause of infertility (male factor alone or male+female factors). All the variables were continuous except for the type of protocol, cause of infertility and smoking status, which were binary variables. Correlation was assessed by the Pearson's correlation coefficients.

The second stage of the analysis was to identify significant predictors of pregnancy. Comparisons were made between pregnant and non-pregnant groups. In multivariable analysis, the Multiple Logistic Regression was used to determine the independent effect of individual variables on clinical pregnancy. First, variables which were significant at P < 0.25 in the univariable analysis were considered as candidates for the multiple logistic regression model, to minimize erroneous exclusion of factors of prognostic relevance. Second, a stepwise approach was applied. Finally, the regression model was calculated only with the covariates that were found to have a significant effect (P < 0.05) on any of the first two steps. Adjusted odds ratio and 95% Confidence Intervals (CI) resulting from the final model were estimated for all the factors remaining in the model.

For both multiple logistic regression and multiple regression models, the stepwise procedure deletes subjects with missing data for any variable which is considered in the model, so that once the final set of variables associated with the outcome was identified, the model was rerun using only the significant variables. This method ensures that the final model is based on the largest possible sample size. Both forward and backward selection methods were used to obtain the smallest number of explanatory variables that provided a well-fitting model. For both multiple logistic regression and multiple regression models, the continuous variable modelling was tested by the fractional polynomial method, a procedure that makes use of the full information available in the data when a linear relationship is not assumed [35]. This approach was used for finding the best fitting functional form between the response variable and one or more continuous covariates, after considering a set of possible transformations [36]. Specific interactions between parameters of interest were also investigated. The area under the Receiver Operating Characteristic (ROC AUC) curve was computed to assess the predictive accuracy of the logistic model, yielding values from 0.5 (no predictive power) to 1.0 (perfect prediction). The Hosmer-Lemeshow goodness of fit test was used to check for lack of fit of the final logistic model. All statistical tests were two-tailed and a P value lower than 0.05 was considered to be statistically significant. Statistical analyses of the data were performed with SPSS, version 15.0 and STATA, version 9.0.

## Results

A total of 278 ICSI cycles were included in the study after controlled ovarian hyperstimulation using a GnRH analogue, 123 with an agonist (44.2%) and 155 with antagonists (55.8%). Female age (range: 23–44 years) group distribution was 26.3% (≤ 29 years), 39.2% (30–34 years), 24.8% (35–39 years) and 9.7% (≥ 40 years). Of the women, 61 (21.9%) were smokers and 217 (78.1%) non-smokers. Causes of infertility were identical between the two stimulation protocol groups, with 65.5% being due to male factor only, 4.7% female factor only, 28.1% male+female factors and 1.8% idiopathic. Cumulatively, 93.5% were due to male factor, 14.7% ovulation disorders, 7.2% endometriosis, 6.8% uterine factor, 5.0% tubar factor and 2.2% other female factors. Embryo transfer was carried out in 270 (97.1%) patients. Lack of transfer was caused by insufficient ovarian response (n = 2), fertilization failure (n = 3), risk of ovarian hyperstimulation syndrome (n = 2) and poor-quality embryos for transfer (n = 1). The pregnancy rates per 270 embryo transfers were 35.9% (97 biochemical), 31.1% (84 clinical) and 27.8% (75 term). Thus, there was a 22/97 (22.7%) rate of embryo loss, 13/97 (13.4%) from biochemical to clinical pregnancy and 9/84 (10.7%) from clinical to term pregnancy. The latter was due to 1/84 (1.2%) ectopic pregnancy and 8/84 (9.5%) first trimester spontaneous abortions. Of the clinical pregnancies, 72.6% were singletons and 25% twins, giving an implantation rate of 18.2%. Although no women were admitted to the hospital, 21 (7.6%) had mild/moderate ovarian hyperstimulation syndrome (OHSS). Table 1 summarizes the demographic data and ovarian responses. Patient baseline characteristics were identical between the two treatment groups with the exception of a significant lower duration of GnRH analogue (4.3 ± 1.2 vs. 27.6 ± 7.0, P < 0.001) and rFSH (8.9 ± 1.3 vs. 11.1 ± 3.0, P < 0.001) treatment in the antagonist group. Laboratorial data showed in the antagonist group a significant higher number of retrieved cumulus-oocyte complexes (11.1 ± 6.0 vs. 8.0 ± 4.5, P < 0.001), metaphase II oocytes (9.0 ± 5.1 vs. 6.7 ± 3.8, P < 0.001) and high quality embryos (5.3 ± 3.9 vs. 3.2 ± 2.5, P < 0.001), and a higher embryo cleavage rate (98% vs. 96%, P = 0.027), whereas the agonist group exhibited a significant higher fertilization rate (79.2% vs. 71.3%, P < 0.001) and a relative higher number of transferred embryos (2.4 ± 0.7 vs. 2.1 ± 0.6, P = 0.001). No significant differences between groups were found regarding clinical outcome variables, with 16.1% vs. 20.6% of implantation rate, 34.8% vs. 36.5% of biochemical, 27.8% vs. 35.3% of clinical, and 26.5% vs. 29.4% of term pregnancy rate per transfer, respectively for women subjected to an antagonist or agonist protocol.

**Table 1:** Summary of demographic data and ovarian responses.

**Parameters**	
**Number of cycles**	278
**Female age (years)**	32.8 ± 4.7
**Duration of infertility (years)**	4.7 ± 3.4
**Type of infertility**	
Primary (never pregnant)	237 (85.3)
Secondary	41 (14.7)
**Number of attempts**	1.6 ± 1.0
**Basal FSH (mIU/ml)**	6.4 ± 2.4
**Basal E2 (pg/ml)**	60.3 ± 58.7
**BMI (Kg/m^2^)**	23.0 ± 3.5
**Ovarian volume (cm^3^)**	
Right	6.4 ± 4.3
Left	6.0 ± 3.8
Total	12.3 ± 6.4
**Total number of antral follicles**	
Right	3.1 ± 2.1
Left	3.0 ± 2.0
Total	6.0 ± 3.8
**Total rFSH administered (IU)**	2139.2 ± 930.6
**Duration of rFSH administration (days)**	9.9 ± 2.5
**Number of follicles > 17 mm**	4.4 ± 2.1
**Number of oocytes**	9.7 ± 5.6
**Number of metaphase II oocytes**	8.0 ± 4.7
**Number of fertilized oocytes**	5.9 ± 3.7
**Number of cleaved embryos**	5.8 ± 3.6
**Number of transferred embryos**	2.2 ± 0.7
**Endometrial thickness (mm)**	10.3 ± 1.6
**Serum E2 on hCG administration day (pg/ml)**	1944.4 ± 1135.1

Data in N, N (%) or mean ± SD.

### Predictors of the number of retrieved oocytes

Ten predictor variables were considered with the dependent variable being the number of retrieved oocytes (age, type of protocol, basal estradiol and FSH levels, total number of antral follicles, ovarian volume, BMI, smoking status, number of attempts, cause of infertility, male or male+female). Only five factors were found significant in the univariable analysis: female age and basal FSH were variables inversely related to the number of retrieved oocytes, whereas the total ovarian volume and total number of antral follicles had a positive correlation to the ovarian response. The patients from the antagonist protocol had a larger number of oocytes retrieved than patients treated with the agonist protocol (Table 2).

**Table 2:** Significant predictors of the number of retrieved oocytes and high quality embryos by linear regression.

	**Univariable analysis**			**Multivariable analysis**		
**Parameters**	**R**^2^	**Unstandardized coefficients**	***P *value**	**Unstandardized coefficients**	**Standardized coefficients**	***P *value**
**Number of retrieved oocytes**						
Protocol (a)	0.072	3.030	< .001	2.667	0.237	< .001
Female age (b)	0.067	- 1.556	< .001	- 0.854	- 0.142	.010
Basal FSH (mIU/ml)	0.079	- 0.656	< .001	- 0.343	- 0.147	.009
Total ovarian volume (cm^3^)	0.026	0.141	.007			
Total number of antral follicles	0.141	0.554	< .001	0.459	0.311	< .001
**Number of high quality embryos (c)**						
Protocol (a)	0.074	0.379	< .001	0.345	0.247	< .001
Female age (b)	0.071	- 0.199	< .001	- 0.136	- 0.182	.002
Basal FSH (mIU/ml)	0.068	- 0.076	< .001	- 0.048	- 0.165	.005
Total ovarian volume (cm^3^)	0.015	0.013	.045			
Total number of antral follicles	0.041	0.037	.001	0.024	0.132	.022

(a) Agonist protocol as reference. (b) For a 5 year increase. (c) Data was log transformed: ln (number of high quality embryos + 1).

Negative coefficients for inversely related variables. Number of retrieved oocytes: model accounts for 25% variability of the number of retrieved oocytes. Number of high quality embryos: model accounts for 17% variability of the number of high quality embryos.

After adjusting for the effects of a large set of variables, multiple regression analysis showed that the total number of retrieved oocytes could be predicted by the female age, type of protocol, basal FSH and total number of antral follicles, with all other parameters being excluded from the equation. Although the ovarian volume was a significant predictor of the number of retrieved oocytes in the univariable analysis, it was not an independent predictor in the multiple regression model. This could occur because the ovarian volume was significantly correlated with the dependent variables basal FSH serum levels (R = -0.130, P = 0.030) and total number of antral follicles (R = 0.219, P < 0.001). The results obtained with this stepwise procedure were also compared with both backward and forward selection methods to identify the smallest number of explanatory variables that provided a well-fitting model. The possible interactions were examined but none of these were significant. We also checked the correct form for continuous variables in the model, using the fractional polynomial method. None of the alternative models was however significantly better than the one considering the linearity of variables. From the multivariable model, the calculated formula to estimate the number of retrieved oocytes was:

Total (COC) = 13.270 + 2.667(GnRH) - 0.854(FA/5) -0.343(FSH) +0.459(TAF)

with

GnRH (agonist = 0; antagonist = 1), FA (female age), FSH (basal FSH), and TAF (Total Antral Follicles).

### Predictors of the number of high quality embryos attained

Once only cycles with transfer of high quality (grade A/B) embryos resulted in pregnancy (21 cycles without pregnancy after transfer of only low quality embryos), we identified those factors that affected the number of high quality embryos attained. Data was log-transformed to achieve the residual normality required in linear regression [ln (number of high quality embryos + 1)], as the number of high quality embryos could take the zero value. First, a univariable analysis was made using the same group of variables studied for the total number of oocytes retrieved, as well as sperm concentration, rapid progressive motility and normal morphology. This revealed the same significant predictors as above. Female age and basal FSH were inversely related to the number of high quality embryos, whereas the total ovarian volume and the total number of antral follicles had a positive correlation. Patients from the antagonist group tended to have a larger number of high quality embryos than patients treated with the agonist protocol (Table 2). Using the multiple regression model, we determined the set of factors that independently predicted the number of high quality embryos. Female age and basal FSH remained significant risk factors for reduced high quality embryo numbers, and elevations in the total number of antral follicles tended to increase the number of high quality embryos. Belonging to the antagonist protocol group was associated with a higher number of high quality embryos. From the multivariable model, the calculated formula to estimate the number of high quality embryos was:

ln(total AB embryos + 1) = 2.316 + 0.345(GnRH) - 0.136(FA/5)- 0.048(FSH) + 0.024(TAF)

with

GnRH (agonist = 0; antagonist = 1), FA (female age), FSH (basal FSH), and TAF (Total Antral Follicles).

### Predictors of pregnancy

The analysis was extended to identify significant predictors of pregnancy. Comparisons of baseline characteristics and ovarian response data between pregnant and non-pregnant groups showed that in the pregnant group the patients were significantly younger, had a lower duration of infertility, fewer number of attempts, lower basal FSH levels, lower total dose of rFSH administered, higher numbers of mature and fertilized oocytes, higher numbers of embryos and higher endometrial thickness (Table 3). The multiple logistic regression model with the dependent variable being the occurrence of clinical pregnancy was restricted to cycles with transfer of high quality embryos and therefore the model is only valid for those cases. Women without transfer of high quality embryos had no pregnancy and this could lead to a numerical problem in statistics. The agonist protocol, less number of attempts, each additional millimetre of endometrial thickness and female age were independently and significantly associated with achievement of clinical pregnancy (Table 4). The possible interactions were examined but none of these were significant.

**Table 3:** Comparison of baseline data and cycle characteristics in ICSI cycles between pregnant and non-pregnant patients (clinical pregnancy).

**Parameters**	**Pregnant**	**Non-Pregnant**	***P *value**
**Number of cycles**	84	186	
**Female age (years)**	31.4 ± 3.8	33.3 ± 4.8	.006
**Duration of infertility (years)**	3.9 ± 2.7	4.9 ± 3.5	.018
**Type of infertility**			.202
Primary (never pregnant)	75 (89.3)	154 (82.8)	
Secondary	9 (10.7)	32 (17.2)	
**Type of protocol**			.233
Antagonist	42 (50.0)	109 (58.6)	
Agonist	42 (50.0)	77 (41.4)	
**Number of attempts**	1.3 ± 0.8	1.6 ± 1.1	.001
**Basal FSH (mIU/ml)**	5.8 ± 1.9	6.6 ± 2.5	.025
**Basal E2 (pg/ml)**	54.8 ± 48.6	61.6 ± 63.0	.367
**BMI (Kg/m^2^)**	23.1 ± 3.7	22.9 ± 3.4	.846
**Total ovarian volume (cm^3^)**	13.4 ± 7.8	11.9 ± 5.7	.252
**Total number of antral follicles**	6.4 ± 4.1	6.0 ± 3.7	.694
**Initial FSH dose (IU)**	190.8 ± 53.3	212.2 ± 66.6	.009
**Total rFSH administered (IU)**	1961.3 ± 884.3	2210.7 ± 928.7	.012
**Duration of rFSH administration (days)**	9.8 ± 2.2	10.0 ± 2.6	.503
**Number of follicles ≥ 17 mm**	4.4 ± 2.1	4.3 ± 2.0	.765
**Number of retrieved oocytes**	10.2 ± 4.6	9.6 ± 5.8	.070
**Number of metaphase II oocytes**	8.7 ± 4.2	7.8 ± 4.7	.031
**Number of fertilized oocytes**	6.7 ± 3.5	5.7 ± 3.6	.013
**Number of cleaved embryos**	6.5 ± 3.4	5.5 ± 3.6	.008
**Number of transferred embryos**	2.2 ± 0.7	2.2 ± 0.7	.958
**Embryo transfer day**			1.000
Day 3	56 (66.7)	124 (66.7)	
Day 5	28 (33.3)	62 (33.3)	
**Number of high quality embryos transferred**			.802
1	12 (14.3)	29 (17.6)	
2	50 (59.5)	94 (57.0)	
3	22 (57.8)	42 (25.5)	
**Endometrial thickness (mm)**	10.6 ± 1.6	10.2 ± 1.5	.038
**Serum E2 on hCG administration day (pg/ml)**	1885.3 ± 1009.8	1963.0 ± 1168.4	.803

Data in N, N (%) or mean ± standard deviation.

**Table 4:** Significant predictors of clinical pregnancy by multiple logistic regression.

	**Adjusted OR (b)**	**CI 95%**	***P *value**
**Type of protocol**			
Agonist (a)	1		.045
Antagonist	0.561	0.318 – 0.988	
**Number of attempts**	0.635	0.430 – 0.936	.022
**Endometrial thickness (mm)**	1.209	1.010 – 1.447	.039
**(Female age/10)**^3^	1.677	1.058 – 2.657	.028
**(Female age/10)^3 ^× ln (Female age/10)**	0.701	0.518 – 0.948	.021

(a) Reference category. (b) Odds Ratios < 1 are associated with decreased probability of pregnancy.

**Figure 1:**
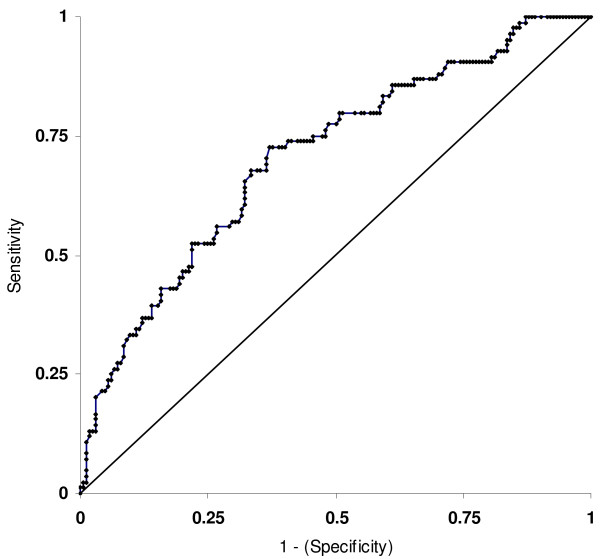
**ROC curve for multiple logistic regression model**. The diagonal line is the line of no discrimination.

Fractional polynomial modelling confirmed that the association with age was not linear. The variable age was thus represented in the model by two terms, (Age/10)^3 ^and (Age/10)^3 ^× ln (Age/10), to properly represent its functional relationship with the probability of getting pregnant as determined by preliminary goodness-of-fit analyses. The interpretation of the variable age is not so simple once it was not included in the model in the linear form, but was modelled by the method of fractional polynomials. To achieve a better understanding, we calculated the estimated adjusted odds ratios for the probability of achieving a clinical pregnancy for female patients of different ages relatively to a 25 years-old patient, considered as the reference, while the other variables in the model were held constant (Table 5). Women 30 years-old were 1.5 times more likely to become pregnant than those with 25 years, 35 years-old women had a similar probability as the reference age, and 40 years-old women had about 4 times less. It seems to be an early increase of the probability to achieve pregnancy until 30 years-old, with a decline after that age and with a sharp decline in women whose ovaries have more than 40 years. To assess the fit of the final logistic model, the Hosmer-Lemeshow test was calculated and demonstrated no lack of fit (P = 0.155). The diagnostic accuracy of the model to discriminate between pregnant and non-pregnant cases was analysed using the ROC curve and AUC_ROC _value (Fig. 1). Its discriminative power was modest (0.707; values vary from 0.5, no predictive power, to 1.0, perfect prediction).

**Table 5:** Estimated odds ratio by female age regarding the occurrence of a clinical pregnancy.

**Female age (years)**	**OR**	**CI 95%**
**25 (a)**	1	
**26**	1.138	0.973 – 1.331
**27**	1.272	0.940 – 1.719
**28**	1.391	0.901 – 2.147
**29**	1.485	0.855 – 2.579
**30**	1.542	0.802 – 2.967
**31**	1.554	0.743 – 3.252
**32**	1.514	0.678 – 3.384
**33**	1.422	0.607 – 3.330
**34**	1.283	0.533 – 3.092
**35**	1.108	0.454 – 2.708
**36**	0.914	0.372 – 2.242
**37**	0.716	0.290 – 1.767
**38**	0.532	0.210 – 1.344
**39**	0.373	0.138 – 1.003
**40**	0.246	0.081 – 0.747
**41**	0.152	0.041 – 0.562
**42**	0.087	0.018 – 0.427
**43**	0.047	0.007 – 0.326
**44**	0.023	0.002 – 0.249

(a) Reference category.

Accordingly (Table 4) the calculated formula to estimate the probability of pregnancy for the given values of the predictive variables in the model was:

Probability of Clinical Pregnancy = e^η^/(1 + e^η^)

where

η = -0.085 - 0.454(NA - 1.528) + 0.190(ET - 10.32) + 0.517 [(FA/10)^3 ^- 34.47] - 0.355 [(FA/10)^3 ^× ln(FA/10) - 40.67] - 0.579(GnRH)

with

NA (number of attempts), ET (endometrial thickness), FA (female age), GnRH (agonist = 0; antagonist = 1).

The estimated probability of clinical pregnancy for hypothetical cases was calculated considering the type of protocol, number of previous attempts, female age and endometrial thickness (Table 6).

**Table 6:** Estimated probability of clinical pregnancy for hypothetical cases.

**Number of attempts**	**Female age (years)**	**Endometrial thickness**			
		**Antagonist**		**Agonist**	
		**9 mm**	**11 mm**	**9 mm**	**11 mm**
**1**	28	32%	41%	46%	56%
	32	34%	43%	48%	58%
	40	8%	11%	13%	18%
**3**	28	16%	22%	26%	34%
	32	17%	24%	27%	36%
	40	3%	5%	6%	8%

## Discussion

Studies comparing the efficacy of the ovarian stimulation with GnRH analogues using univariable analysis [3-7,9] or meta-analysis of randomized controlled trials [14], showed no significant differences in pregnancy rates between antagonist and agonists. In contrast, other meta-analyses of randomized controlled studies [12,13] and reviews based on randomized studies [37,38], have shown that if the antagonist protocol avoids the adverse effects of agonists and is significantly associated with a lower duration of treatment and total administered doses of the GnRH analogue and of rFSH, the agonist regimen appears associated with higher numbers of oocytes and embryos and with higher implantation and pregnancy rates. However, with the exception of a single study, which showed that the chance of pregnancy was significantly improved when a GnRH agonist ultrashort protocol was used in detriment of clomiphene citrate [27], the stimulation protocol has not been included in the multivariable analysis of those studies.

The present study was primarily designed to investigate the predictive value of the stimulation protocol and to analyze the possible relationships between stimulation protocols and treatment outcomes after adjusting for a large set of variables that potentially affect reproductive outcomes. Nevertheless, the present study design has as main limitation the fact of being an observational prospective study and not a randomized controlled trial. However, as the two studied groups were comparable, the possibility of selection appears minimal. We here show that the ovarian stimulation protocol affects the reproductive outcomes, with patients in the antagonist group having lower duration of rFSH administration, higher numbers of retrieved oocytes and high quality embryos, whereas those from the agonist group will present higher fertilization rates.

Regarding predictors of the number of retrieved oocytes and high quality embryos, the present multiple regression analysis showed a positive association with use of the antagonist protocol and the total number of antral follicles, and a negative association with increasing maternal age and basal serum levels of FSH. For the ovarian response, these results are in agreement with others relatively to female age, serum basal FSH and the total number of antral follicles [19-23,26,28,30,39]. The model explained only 25% of the variation of the ovarian response, which is in accordance (25–38%) to other studies [20,22,23,28], and 17% of the variability of the number of high quality embryos. These values strongly suggest that variability may be due to inherent biological mechanisms as also to parameters that may not be considered or only partially controlled, particularly genetic factors [40].

The present study also demonstrated that the likelihood of pregnancy is positively associated with use of the agonist stimulation protocol, less number of attempts and higher endometrial thickness. The likelihood of getting pregnant was also shown to increase up to age 30, decreasing afterwards, with a sharp decline for women above 40 years-old. Regarding female age, number of attempts and endometrial thickness, our present results are in agreement with previous studies [16,21,24-27,29,30,33,41,42]. However, in contrast with other studies, higher serum basal levels of FSH and estradiol, longer duration of infertility, female smoking, combined male and female factor infertility or multiple female infertile factors were not found as significant negative predictors of pregnancy [16,19,21,25,26,30,31,42]. Similarly, the present data also do not support idiopathic infertility, ovulation dysfunction other than diminished ovarian reserve, higher serum levels of estradiol on the day of hCG injection, and higher numbers of oocytes, embryos, high quality embryos and transferred embryos as significant positive predictors of pregnancy achievement [21,24,25,27,29,30].

Although the pregnancy rates did not differ between both protocols, the multiple logistic regression analysis confirmed a marginal but significant higher probability of achieving a clinical pregnancy in the agonist group (OR = 0.561; 0.318–0.988; P = 0.045). Because the antagonist group had a higher loss of early implanted embryos (biochemical to clinical pregnancy, 22.2% vs. 2.3%) and a lower loss of well implanted embryos (clinical to term pregnancy, 4.8% vs. 16.7%), the lower probability of a successful clinical pregnancy after antagonist treatment might be associated with endometrial characteristics or the genetical profile of the oocytes and embryos [10,43].

In conclusion, the present study analyzed the impact of the stimulation protocol on reproductive outcomes adjusting for an extended number of potential risk factors, providing useful information about the independent predictors of ovarian response, production of high quality embryos and occurrence of a clinical pregnancy. Quantitative data showed that the antagonist protocol is associated with more oocytes and high quality embryos than the agonist. Although it seemed to be a trend towards higher pregnancy rates in the agonist group, this association was marginal (P = 0.045), not allowing an evident distinction of benefits between each protocol on achieving a pregnancy. Data also showed that not only the protocol affected the outcomes but also factors as female age, serum basal levels of FSH, total number of antral follicles, number of previous treatment attempts, and endometrium thickness should be analysed when the purpose is to choose a therapy and assess the chance of success for better counselling patients before undergoing an ICSI treatment.

## Competing interests

The authors declare that they have no competing interests.

## Authors' contributions

FP was responsible for data collection, data analysis, data interpretation and writing of the manuscript. CO was responsible for female clinical evaluation and treatment, and reviewed the manuscript. JTS participated in female clinical evaluation and treatment, and reviewed the manuscript. MFC designed and supervised the statistical work, and participated in data analysis, data interpretation and writing of the manuscript. JS was responsible for the IVF work and reviewed the manuscript. AB was responsible for recruitment of the patients, spermiograms, genetical studies and supervision of IVF treatments, and reviewed the manuscript. MS conceived, designed and coordinated the study, participated in data interpretation, and wrote the final manuscript.

All authors read and approved the final manuscript.
